# The metabolic dysfunction-associated steatohepatitis (MASH) drug resmetirom exhibits broad nuclear receptor activity with minimal functional impact

**DOI:** 10.1038/s41598-026-37494-y

**Published:** 2026-01-29

**Authors:** Annette Kärcher, Laura Isigkeit, Nils Christiaan Bandomir, Manfred Schubert-Zsilavecz, Pascal Heitel

**Affiliations:** 1 Goethe University Frankfurt, Institute of Pharmaceutical Chemistry, Max-Von-Laue-Str. 9, 60438 Frankfurt, Germany; 2 Goethe University Frankfurt, Institute of Pharmacology & Clinical Pharmacy, Max-Von-Laue-Str. 9, 60438 Frankfurt, Germany; 3 Georg-Speyer-Haus, Paul-Ehrlich-Str. 42-44, 60596 Frankfurt, Germany

**Keywords:** Nuclear receptor modulator, Metabolic syndrome, Reporter gene assay, Target gene expression, Thyroid hormone receptor β, Diseases, Drug discovery, Endocrinology, Gastroenterology, Medical research

## Abstract

In 2024, the FDA granted approval for resmetirom, a selective but moderately potent agonist of the thyroid hormone receptor (THR) β, marking the first drug for the treatment of metabolic dysfunction-associated steatohepatitis (MASH) in the US. Given the absence of selectivity data, we conducted a comprehensive screening against other nuclear receptors implicated in metabolic regulation, in addition to THRβ. Reporter gene assays revealed that resmetirom also binds several off-target nuclear receptors, including constitutive androstane receptor (CAR), retinoic acid receptor-related orphan receptors (RORs), and hepatocyte nuclear factor 4α (HNF4α). However, subsequent in vitro experiments, designed to better recapitulate physiological conditions, showed that THRβ modulation is functionally dominant. These preliminary findings suggest that pharmacotherapeutic efficacy of resmetirom remains intact despite the identified off-target effects, supporting its clinical application in the treatment of MASH.

**Supplementary Information:**

The online version contains supplementary material available at 10.1038/s41598-026-37494-y.

## Introduction

The prevalence of metabolic dysfunction-associated steatohepatitis (MASH), formerly known as non-alcoholic steatohepatitis (NASH), is increasing dramatically around the world^1^. Although precise figures are difficult to determine because MASH diagnosis currently requires a liver biopsy, it is estimated that around 5% of the world population suffer from MASH^1^. MASH is a severe form of metabolic dysfunction-associated steatotic liver disease (MASLD), which is characterized by hepatic steatosis. The excessive accumulation of triglycerides in the liver can cause lipotoxicity and inflammation so that an estimated 30% of MASLD patients develop MASH^1,2^. Overnutrition and lack of physical exercise are key drivers in the complex MASH pathogenesis^3^. If untreated, MASH can lead to progressive fibrosis, cirrhosis and hepatocellular carcinoma (HCC)^2,3^.

In March 2024, the selective thyroid hormone receptor (THR) β agonist resmetirom has become the first MASH drug to gain FDA approval, marking a milestone in MASH pharmacotherapy^4,5^. In August 2025, the glucagon-like peptide 1 (GLP-1) receptor agonist semaglutide followed resmetirom and was also granted approval by the FDA, highlighting the great interest in MASH therapy.

**Fig. 1:**
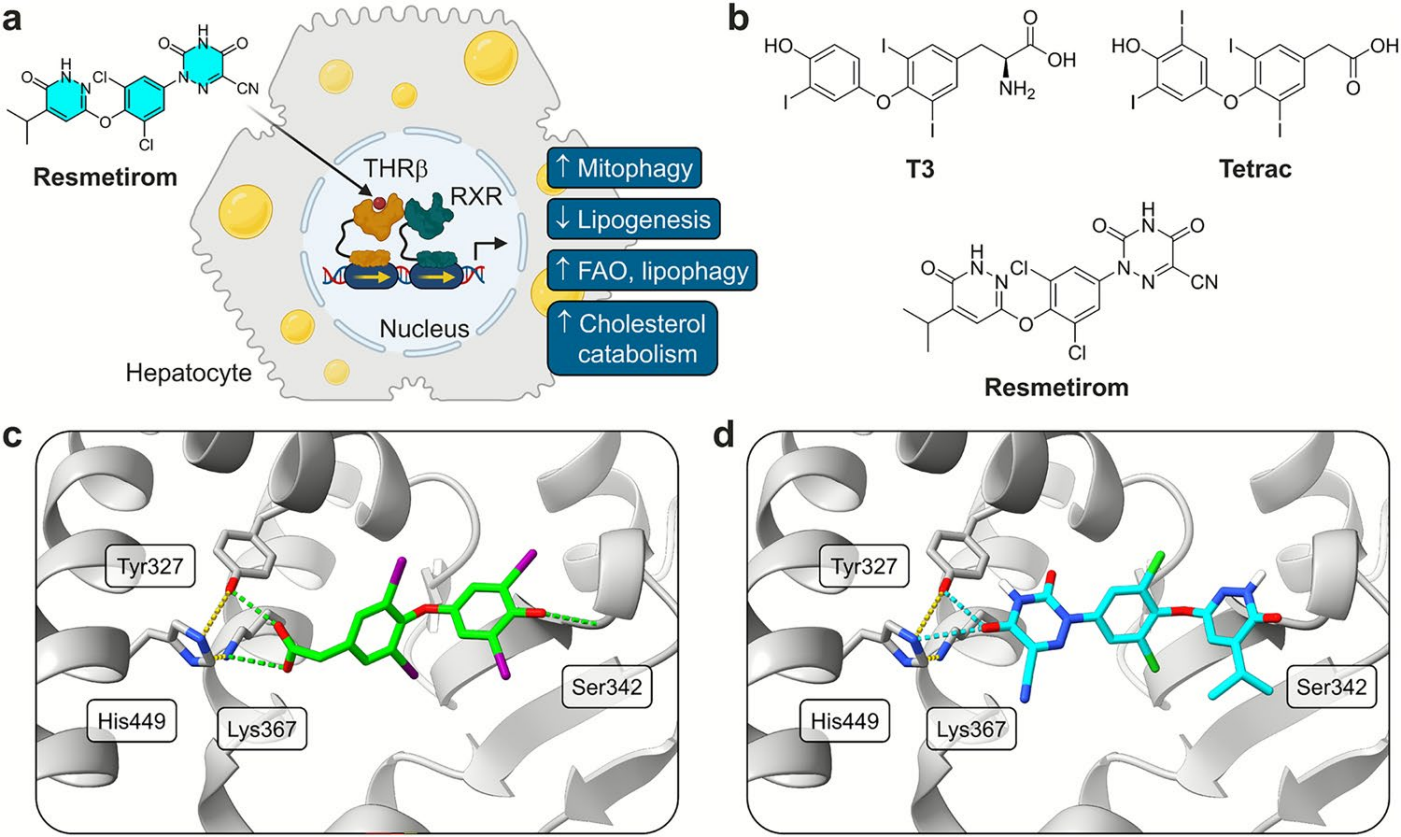
Mode of action and structure of resmetirom. (**a**) Resmetirom is a selective agonist for the nuclear receptor THRβ. This transcription factor regulates a set of genes in hepatocytes with a plurality of effects, including decreased de novo lipogenesis, increased lipophagy and fatty acid oxidation. FAO, fatty acid β oxidation; THR, thyroid hormone receptor; RXR, retinoid X receptor. Figure created with BioRender. (**b**) Chemical structure of resmetirom, the thyroid hormone triiodothyronine (T3), and the thyroid hormone metabolite Tetrac. (**c**) Co-crystal structure of Tetrac (green) in the ligand binding domain of peroxisome proliferator-activated receptor γ (PPARγ, PDB ID 6tsg^13^). Tetrac forms a hydrogen bond network with Tyr327, a salt bridge with Lys367, and interacts with the protein backbone of Ser342. Intramolecular hydrogen bonds are colored yellow. (**d**) Molecular docking of resmetirom (cyan) in PPARγ (PDB ID 6tsg^13^) suggested a distinct binding mode with hydrogen bonds to Tyr327 and His449, but not to Ser342. Intramolecular hydrogen bonds are colored yellow.

THR is a ligand-activated transcription factor of the nuclear receptor superfamily, that plays a pivotal role in the physiological action of the thyroid hormones triiodothyronine (T3) and tetraiodothyronine (T4).^6^ THR regulates a multitude of biological processes including energy metabolism, growth and development, heart rate, cardiac function, and body temperature^6,7^. THR commonly forms non-permissive heterodimers with retinoid X receptor (RXR, NR2B1-3) i.e., they can be activated by THR ligands (such as T3) alone, but not by RXR ligands alone (Fig. 1a)^6^. The presence of both ligands causes synergistic activity on target gene transcription. Activation of THR by T3 leads to the transcription or repression of specific target genes (negative regulation by liganded THRs is not yet understood^8^), thus accounting for the multitude of physiological functions that depend on thyroid hormones^6^. There are two receptor subtypes, THRα (NR1A1) and THRβ (NR1A2) with different expression patterns. While THRα is the major isoform in the heart and bone, THRβ is dominant in the liver^9^.

Euthyroid MASH patients have been found to suffer from intrahepatic hypothyroidism, which is characterized by reduced conversion of T4 to the more effective T3 and increased formation of the inactive T4 metabolite reverse T3^10,11^. This leads to an impaired THR signaling in the liver and has been associated with the promotion of MASH progression^10,11^. Resmetirom has been developed to selectively activate the THRβ subtype and to act in the liver to avoid cardiac or other peripheric side effects such as bone loss^5,6,9^. The THRβ agonist resmetirom enhances fatty acid β oxidation, lipophagy, mitophagy, and cholesterol catabolism in the liver, while inhibiting de novo lipogenesis^12^. In clinical trials, the treatment with resmetirom has proven to lower low-density lipoprotein (LDL) cholesterol and triglyceride levels, to improve fibrosis scores, and to resolve MASH in patients with liver fibrosis^12^.

Given that T3 and T4 metabolites, such as tetraiodothyroacetic acid (Tetrac or TA4, Fig. 1b), have been shown to modulate the peroxisome proliferator-activated receptor γ (PPARγ, NR1C3) from the same nuclear receptor subfamily as THR^13^, we were prompted to investigate whether resmetirom also binds other nuclear receptors. Considering the absence of information on nuclear receptor selectivity in the literature, we explored the effects of resmetirom on PPARs and a selected panel of nuclear receptors associated with metabolic processes. In this study, we present our findings on the activity of resmetirom on a selection of off-target nuclear receptors. Our preliminary investigations suggest that the off-target activities do not compromise the pharmacological effects of resmetirom’s THRβ agonism.

## Results

### Molecular docking

Due to its structural similarity to T3, resmetirom is categorized as an aryloxyphenyl-based thyromimetic (Fig. 1b)^5,9^. It also resembles the T3 metabolite Tetrac, which is a PPARγ agonist with nanomolar EC_50_ value and, to a lesser extent, also activates RXRs^13^. In a first step, we performed a molecular docking simulation to predict whether resmetirom also binds PPARγ. Comparison of the molecular docking pose of resmetirom in the PPARγ ligand binding pocket with the published co-crystal structure of Tetrac in PPARγ (PDB ID 6tsg^13^) however suggested distinct binding modes (Fig. 1c,d). The co-crystallized Tetrac shows a strong stabilization of the PPARγ ligand binding domain, with its carboxylate moiety forming a hydrogen bond to Tyr327 and a salt bridge to Lys367. The phenol group on the opposite end of the molecule is engaged in an additional hydrogen bond to the amide backbone of Ser342. The core tetraiododiphenylether motif of Tetrac occupies a lipophilic region within the PPARγ ligand binding pocket and interacts via van der Waals forces. Overall, it exhibits a unique binding mode characterized by bilateral terminal stabilization of the PPARγ ligand binding domain using hydrogen bonds and ionic interactions.

In contrast, molecular docking of resmetirom using AutoDock Vina^14^ (Fig. 1d) suggested that the key interactions to PPARγ are a hydrogen bond network formed with Tyr327 and His449. These amino acids build a basic triad together with Lys367 that is stabilized by hydrogen bonds. While both Tetrac and resmetirom engage with this basic region, only Tetrac, but not resmetirom, forms a hydrogen bond to Ser342 at the other end of the ligand binding pocket. Additionally, the *iso*-propyl group of resmetirom protrudes from the ligand binding pocket and points towards the hydrophilic solvent, which is unfavorable due to the lipophilic nature of this substituent (Figure S1). In summary, despite the structural similarity to Tetrac, molecular docking indicated that resmetirom binds to PPARγ with either less affinity or not at all.

### Reporter gene assay

**Fig. 2:**
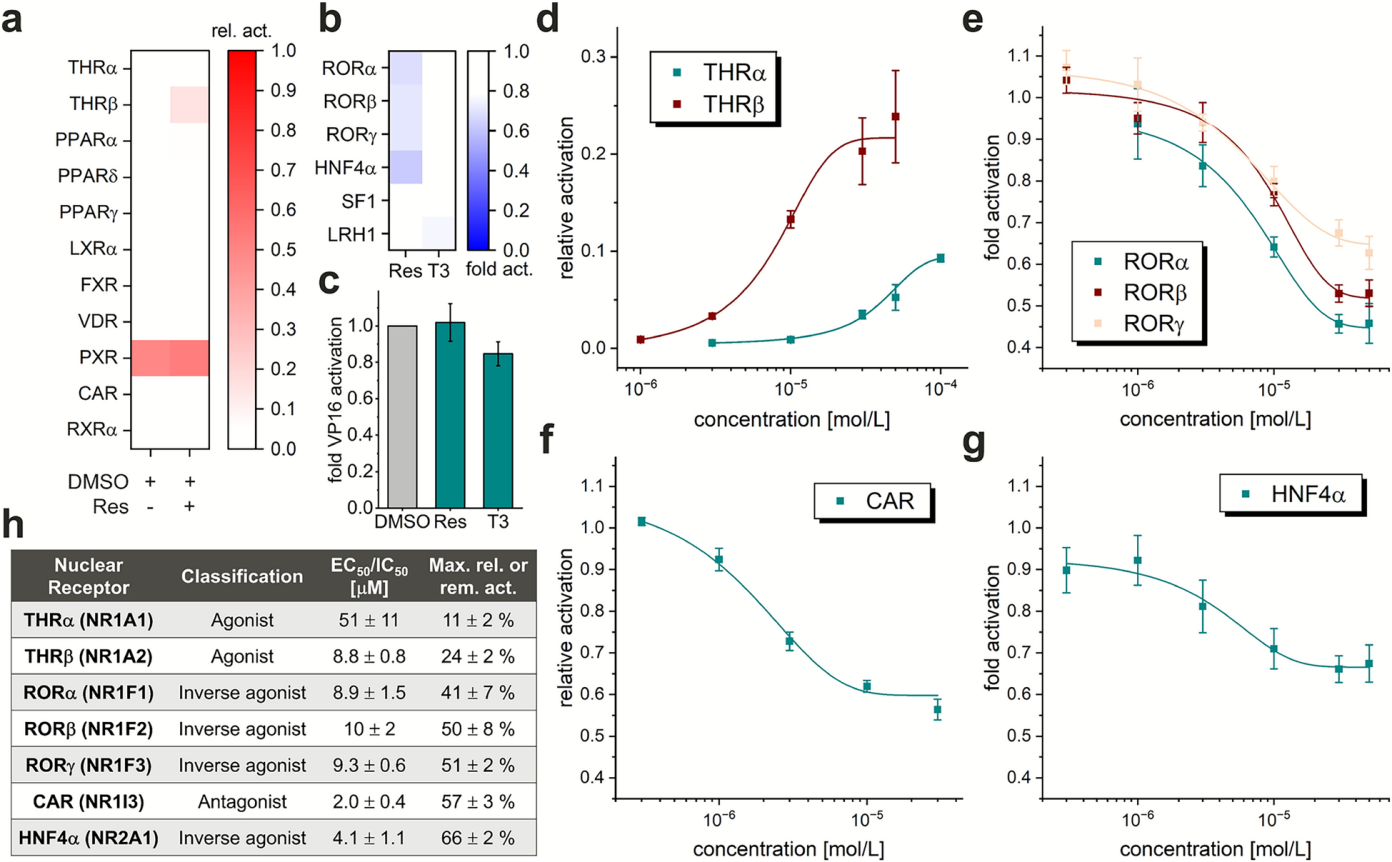
Screening of resmetirom (10 μM) on nuclear receptors involved in diverse metabolic processes. (**a**,**b**) In Gal4 reporter gene assays, (**a**) resmetirom selectively activated THRβ within the panel of ligand-activated nuclear receptors and (**b**) acts as an inverse agonist for the nuclear receptors RORα/β/γ and HNF4α with constitutive activity. The dual THR agonist T3 (10 μM) was inactive on these receptors; *N* = 3; Panel (**a**) shows the relative activation to literature agonists specified in the materials & methods section. As xenobiotic sensor, PXR has a high basal activation. Panel (**b**) shows the fold activation compared to the 0.1% DMSO control. (**c**) Control experiment with the ligand-independent transcriptional activator Gal4-VP16^15^, whose activity was not influenced by resmetirom at 30 μM and T3 at 10 μM. (**d**–**g**) Dose–response curves for resmetirom on THRs (**d**), RORs (**e**), CAR (**f**), and HNF4α (**g**) in the Gal4 reporter gene assay; *N* ≥ 3. (**h**) Pharmacodynamic properties of resmetirom derived from the reporter gene assays.

To evaluate the docking results experimentally, we screened resmetirom on PPARs and closely related nuclear receptors associated with metabolic processes in specific Gal4 hybrid reporter gene assays (Fig. 2). In these cellular test systems, a fusion protein of the respective nuclear receptor ligand binding domain and the DNA binding domain of the yeast receptor Gal4 are overexpressed. A firefly luciferase under control of a Gal4 promoter is used as reporter gene and a constitutively expressed renilla luciferase serves as control for normalization of cell count, transfection efficiency and cytotoxicity. As supported by the molecular docking data, we did not observe PPARγ activation by resmetirom at a concentration of 10 μM (Fig. 2a). In a control experiment, resmetirom had no influence on the transactivation of the ligand-independent VP16-Gal4^15^, thus excluding unspecific binding events in the reporter gene assay screening (Fig. 2c). Resmetirom expectedly induced the transactivation of THRβ and furthermore acted as an antagonist of the constitutive androstane receptor (CAR, NR1I3) and inverse agonist of the constitutively active nuclear receptors retinoic acid receptor-related orphan receptor α (RORα, NR1F1), RORβ (NR1F2), RORγ (NR1F3), and hepatocyte nuclear factor 4α (HNF4α, NR2A1) (Fig. 2b). Subsequent dose–response experiments resulted in EC_50_ values of 8.8 ± 0.8 μM for resmetirom on THRβ, whereas THRα was activated less potently and with lower transactivation efficacy (Fig. 2d,h) than the reference T3 (1 μM). With an EC_50_ of 51 ± 11 μM obtained from toxicity-adjusted data (resmetirom demonstrated weak cytotoxic effects to HEK293T cells at concentrations above 50 μM (Figure S2)), resmetirom had a 5.7-fold selectivity for THRβ over the α subtype. On CAR, resmetirom dose-dependently inhibited the activation of reference agonist CITCO at 1 μM (IC_50_ = 2.0 ± 0.4 μM) but only with partial efficacy (Fig. 2f,h). Finally, resmetirom also acted as a partial inverse agonist for RORα (EC_50_ = 8.9 ± 1.5 μM), RORβ (EC_50_ = 10 ± 2 μM), RORγ (EC_50_ = 9.3 ± 0.6 μM), and HNF4α (EC_50_ = 4.1 ± 1.1 μM) with moderate potencies and approximately halved the intrinsic activation of these receptors (Fig. 2e,g,h). Unlike resmetirom, the dual THRα/THRβ agonist T3 has already been shown to be inactive on our panel of ligand-regulated nuclear receptors^13^. Additional screening on constitutively active nuclear receptors for comparison also confirmed the selective THR agonism of T3 (Fig. 2b). Although T3 tended to decrease the intrinsic activation of liver receptor homolog-1 (LRH-1, NR5A2) with low efficacy, this effect did not reach statistical significance (one-sided t-test against 0.1% DMSO control).

### Target gene expression

**Fig. 3:**
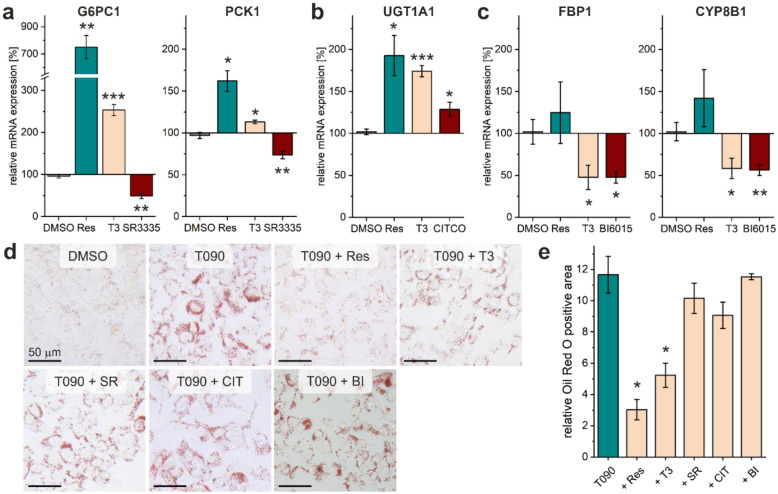
(**a**–**c**) Quantification of THR-, ROR-, CAR- and HNF4α-regulated mRNA expression by qPCR after 24 h in HepG2 cells. While resmetirom significantly induced the expression of shared THR/ROR (**a**) and THR/CAR (**b**) target genes, it had no effect on THR/HNF4α target gene expression (**c**). Data are the mean ± SEM; *N* = 3–4; * p < 0.05, ** p < 0.01, *** p < 0.001 (two-sided t-test against 0.1% DMSO). (**d**,**e**) Lipid-droplet accumulation in HepG2 cells (Oil Red O staining) after 72 h treatment with DMSO (0.1%) control, LXR agonist T0901317 (10 μM, T090) as positive control, and T090 in combination with resmetirom (30 μM), T3 (1 μM), SR3335 (10 μM), CITCO (10 μM), or BI6015 (10 μM). One representative image from three independent experiments is shown (complete set of images available in supplementary information, Figure S3). (**e**) Quantification of Oil Red O positive area relative to the 0.1% DMSO control. Data are the mean ± SEM; *N* = 3; * p < 0.05, (one-sided t-test against 10 μM T090). BI, BI6015; CIT, CITCO; Res, resmetirom; SR, SR3335; T090, T0901317.

To study resmetirom in a physiologically more relevant environment than the artificial reporter gene assays, we determined the expression of target genes in HepG2 cells (hepatocellular carcinoma) by qPCR (Fig. 3a-c). Since THR controls a multitude of target genes, which overlap with the off-target nuclear receptors, we tested T3 for each target in addition to resmetirom and a respective reference ligand. Apart from RORβ, all off-targets are expressed in the liver^16–18^. In HepG2 cells, resmetirom strongly induced mRNA expression of glucose-6-phosphatase catalytic subunit 1 (G6PC1) and phosphoenolpyruvate carboxykinase (PCK1), both of which are key enzymes in gluconeogenesis and ROR target genes (Fig. 3a). While THR agonist T3 also induced the expression of these target genes, the inverse RORα/γ agonist SR3335 had the opposite effect and significantly inhibited G6PC1 and PCK1 mRNA transcription.

Due to the limited commercial availability of selective inverse CAR agonists^19^, we used the agonist CITCO as a reference. The CAR and THR target gene UDP-glucuronosyltransferase 1A1 (UGT1A1) was positively regulated by CITCO, T3, and resmetirom (Fig. 3b). Furthermore, resmetirom did not significantly influence the expression of the HNF4α target genes fructose-1,6-bisphosphatase 1 (FBP1) and cytochrome P450 enzyme 8B1 (CYP8B1, also known as 7-α-hydroxycholest-4-en-3-one 12-α-hydroxylase), while both T3 and the inverse HNF4α agonist BI6015 suppressed these genes (Fig. 3c). There are opposing effects of THRβ agonism and inverse ROR agonism or CAR antagonism, respectively. However, our data suggest that the THRβ-mediated effects dominate in HepG2 cells for G6PC1, PCK1, and UGT1A1, i.e., over the ROR and CAR target genes. In contrast, both the THRβ agonist reference and the inverse HNF4α agonist reference induced the expression of FBP and CYP8B1, but resmetirom had no effects on these genes in our assay setting.

### Lipogenesis assay

Since the THR-mediated effects of resmetirom have dominated the target gene expression, we additionally investigated the influence of off-target nuclear receptors on resmetirom’s ability to inhibit steatosis. For this purpose, we induced lipogenesis in HepG2 cells by treatment with the liver X receptor agonist T0901317 (10 μM) over a period of 72 h (Fig. 3d,e). Co-incubation of T0901317 with the THR agonists resmetirom (30 μM) or T3 (1 μM) inhibited accumulation of neutral lipids as confirmed by Oil Red O staining. In contrast, co-incubation with ROR (SR3335, 10 μM), CAR (CITCO, 10 μM), or HNF4α (BI6015, 10 μM) ligands had no effect on T0901317-induced lipid droplet formation. These data corroborate the assumption that resmetirom’s off-target effects play a negligible role in vitro and therefore do not interfere with the use of resmetirom in MASH pharmacotherapy.

## Discussion

Although resmetirom is an FDA-approved drug for the treatment of MASH, no data have been published regarding its selectivity among nuclear receptors. In Gal4 hybrid reporter gene assays, we confirmed resmetirom as a moderately potent THR agonist with selectivity for THRβ. Literature data reported a higher affinity (THRα: K_d_ = 3.74 μM, THRβ: K_d_ = 0.21 μM) in a co-activator recruitment assay^5^ and a similar potency in a reporter gene assay using Huh7 cells (THRα: EC_50_ = 2.85 μM, THRβ: EC_50_ = 0.99 μM)^20^, but the THRβ selectivity is consistent across all test systems. The discrepancy between our data and the co-activator recruitment assay can be attributed to fundamental differences in the assay design: The literature assay is based on Förster resonance energy transfer (FRET) and measures the proximity of His-tagged THRs to the biotin-labeled co-activator peptide nuclear receptor co-activator 2 (NCoA-2), whereas our assay system is cell-based and not restricted to a specific nuclear receptor co-activator. This is further supported by pharmacokinetic data reporting high plasma binding, mediocre aqueous solubility (1.0 μM at pH 7.04^5^) and low Caco-2 cell permeation^5^, although the same reference claims a liver to plasma ratio of 8:1 in diet-induced obese (DIO) mice.

The recommended dose of resmetirom in MASH patients with moderate to advanced liver fibrosis depends on body weight and is 80 mg (body weight < 100 kg) or 100 mg (body weight ≥ 100 kg) orally once daily, leading to maximum plasma concentrations of 0.78 – 0.97 mg/mL (1.8 – 2.2 μM) at steady state^21^. If the 8:1 liver to plasma ratio translates from DIO mice into humans, the resmetirom concentration (approximately 16 μM) would approximately match the EC_75_ value in our cellular assay system for THRβ, but would not suffice to activate THRα substantially (EC_50_ = 51 μM). There is additional evidence that resmetirom is transported by the liver-specific organic anion transporter OATP1B1 into hepatocytes^22^, which makes a high liver to plasma ratio conceivable. Therefore, the results of our reporter gene assays correlate well with the actual therapeutic doses, which have been selected to avoid THRα activation and its side effects.

However, due to its moderate potency on THRβ (EC_50_ = 8.8 μM), resmetirom requires relatively high clinical doses to achieve a therapeutic effect in the liver. This makes it more susceptible to having undesired side effects than other drugs, which typically achieve pharmacological effects at nanomolar concentrations. Hence, it is not surprising that we characterized RORs, CAR, and HNF4α as nuclear off-targets in the Gal4 hybrid reporter gene assays. In contrast to the weak potency and efficacy on THRα (EC_50_ = 51 μM), the IC_50_ values on these off-target receptors were in a similar range (2 – 10 μM) to the EC_50_ value on THRβ. For this reason, it is generally conceivable that these receptors are inhibited at the estimated concentration in the liver, but presumably not in other tissues. However, the efficacy on off-target nuclear receptors was rather weak, with 41 – 66% remaining constitutive activation in the reporter gene assays. In addition, T3 was completely selective for THRs in our reporter gene assays, strongly indicating that resmetirom’s side activities are compound-specific rather than class-specific.

Due to low off-target efficacy in the reporter gene assays, we evaluated the potential clinical relevance of these results in additional assay systems with less artificial character. In HepG2 cells, resmetirom induced the THR target genes G6PC1^23^, PCK1^24^, which are rate-limiting enzymes in gluconeogenesis, a hallmark of the fasting state. These genes were regulated in the opposite direction by inverse RORα/γ agonist SR3335, so that it can be concluded that the partial agonism on THRβ prevails resmetirom’s inverse agonism on RORs. The same holds true for the CAR target gene UGT1A1^25,26^. As the phase II metabolic enzyme was upregulated by the agonist CITCO, CAR antagonists such as resmetirom are expected to suppress UGT1A1 mRNA expression. However, both resmetirom and T3 induced UGT1A1, indicating that THR-mediated effects could prevail over CAR inhibition. It should be noted that additional validation experiments are required to confirm direct functional competition between CAR antagonism and THR agonism. The failure of resmetirom to suppress UGT1A1 could alternatively be explained by a lack of in vitro efficacy on CAR. The dominance of THR-mediated effects could be the consequence of low inhibition efficacy on both RORs and CAR. This is also consistent with resmetirom’s lack of regulation of HNF4α target genes, where resmetirom had a high remaining activity of 66% in the reporter gene assay.

While resmetirom and T3 suppressed T0901317-induced lipogenesis in HepG2 cells, literature ROR and CAR ligands showed no effect, from which it can be deduced that these off-target receptors are not involved in de novo triglyceride synthesis. As suppressor of the insulin promoter, inverse HNF4α agonist BI6015 has been shown to induce steatosis in murine hepatocytes^27^, and we also observed a moderate lipogenic effect in the Oil Red O assay (Figure S4). However, BI6015 exhibited no additive effects and did not exacerbate T090137-induced lipogenesis. The weak inverse agonistic activity of resmetirom on HNF4α in the reporter gene assay also had the potential to counteract the THR-mediated anti-lipogenic effects. Similar to BI6015, however, resmetirom did not increase lipogenesis in combination with T0901317. Rather, we observed a strong inhibition of the T0901317-induced lipid formation. Again, the off-target activity, weak inverse HNF4α agonism, was repressed by resmetirom’s THRβ agonism.

Nevertheless, the modulation of RORs, CAR, and HNF4α could compromise both the efficacy and long-term safety of MASH therapy. In contrast to RORβ, which is only expressed in retina and brain^28^, RORα, RORγ, CAR, and HNF4α can be found in the liver^16–18^. RORα and RORγ regulate circadian rhythm, lipid and glucose metabolism, and inflammation. Paradoxically, both ROR agonists and inverse agonists have therapeutic potential in MASH. While ROR agonists inhibit oxidative stress and inflammation and negatively regulate PPARγ^29,30^, inverse agonists inhibit hepatic triglyceride synthesis^31^. As a xenobiotic sensor, CAR plays a key role in hepatic detoxification of xenobiotics, but it is also involved in glucose and lipid metabolism. Since there is evidence that CAR activation can have both protective^32^ and detrimental effects^33,34^ in MASH, further research and more selective tool compounds are necessary to establish a clear role of CAR in MASH therapy. HNF4α plays a central and protective role in maintaining liver homeostasis^35^. HNF4α agonists could restore normal liver function in MASH patients^36^. Therefore, inverse agonists like resmetirom could exacerbate an existing MASH, provided that it is able to inhibit HNF4α in vivo.

Combined, our data suggest that the low selectivity of resmetirom among nuclear receptors has no or only little effect in vitro and therefore does not compromise its therapeutic application in MASH with fibrosis. However, the findings of this study have to be seen in light of some limitations. Above all, our results depend on the HepG2 liver carcinoma cell line, which does not fully represent the complexity of in vivo physiology or patient-derived tissues. Further validation in animal models and clinical samples will be essential to confirm these findings.

In conclusion, we report off-target activities of the recently FDA-approved MASH drug resmetirom on several nuclear receptors. Moderate on-target potency (EC_50_ = 8.8 ± 0.8 μM on THRβ) increases the risk of off-targets, and we have identified RORs, CAR, and HNF4α as such. While resmetirom had similar potencies on these receptors as on THRβ in reporter gene assays, the efficacy was only moderate. The further evaluation of resmetirom in target gene expression and lipid accumulation assays provided evidence that the off-target effects are inferior to THR modulation. Our in vitro data suggest that the lipid-lowering properties of resmetirom exploited in MASH therapy are not compromised by other nuclear receptors. Nevertheless, due to its moderate cellular potency on THRβ, there is a high probability that other off-targets beyond nuclear receptors will be addressed by resmetirom. Our research results and the increasing prevalence of MASH underscore the need for novel THRβ-selective agonists with high potency and selectivity.

## Materials & methods

### Tissue culture

HEK293T and HepG2 cells (German Collection of Microorganisms and Cell Cultures, DSMZ) were grown in Dulbecco’s modified Eagle’s medium (DMEM) high glucose supplemented with 10% fetal bovine serum (FBS), sodium pyruvate (1 mM), penicillin (100 U/mL), and streptomycin (100 μg/mL) at 37 °C and 5% CO_2_.

### Plasmids

For the hybrid reporter gene assays, the previously reported Gal4-fusion receptor plasmids pFA-CMV-hTHRα-LBD^13^, pFA-CMV-hTHRβ-LBD^13^, pFA-CMV-hPPARα-LBD^37^, pFA-CMV-hPPARγ-LBD^37^, pFA-CMV-hPPARδ-LBD^37^, pFA-CMV-hRORα-LBD^38^, pFA-CMV-hRORβ-LBD^38^, pFA-CMV-hRORγ-LBD^38^, pFA-CMV-hLXRα-LBD^39^, pFA-CMV-hFXR-LBD^40^, pFA-CMV-hVDR-LBD^41^, pFA-CMV-hPXR-LBD^41^, pFA-CMV-hCAR-LBD^41^, pFA-CMV-hHNF4α-LBD^42^, pFA-CMV-hRXRα-LBD^41^, pFA-CMV-hSF1-LBD^43^, and pFA-CMV-hLRH1-LBD^44^, coding for the hinge region and LBD of the canonical isoform of the respective nuclear receptor were used. pFR-Luc (Stratagene) was used as reporter plasmid and pRL-SV40 (Promega) was used in all assays for normalization of transfection efficiency and cell growth. pECE-SV40-Gal4-VP16 was a gift from Lea Sistonen (Addgene plasmid #71728^15^).

### Reporter gene assays

The day before transfection, HEK293T cells were seeded in 96-well plates (4 × 10^4^ cells/well). Before transfection, the medium was changed to Opti-MEM without supplements. Transient transfection was carried out using Lipofectamine LTX reagent (Invitrogen) according to the manufacturer’s protocol with pFR-Luc (Stratagene), pRL-SV40 (Promega), and the corresponding Gal4-fusion nuclear receptor plasmid. Five hours after transfection, medium was changed to Opti-MEM supplemented with penicillin (100 U/mL), streptomycin (100 μg/mL), and now additionally containing 0.1% DMSO and the respective test compound or 0.1% DMSO alone as untreated control. Each concentration was tested in duplicates, and each experiment was repeated in at least three biologically independent repeats. Following overnight (14 – 16 h) incubation with the test compounds, cells were assayed for luciferase activity using Dual-Glo™ Luciferase Assay System (Promega) according to the manufacturer’s protocol. Luminescence was measured with a Spark 10 M luminometer (Tecan Deutschland GmbH). Normalization of transfection efficiency and cell growth was done by division of firefly luciferase data by renilla luciferase data multiplied by 1,000 resulting in relative light units (RLU). Fold activation was obtained by dividing the mean RLU of the tested compound at a respective concentration by the mean RLU of the untreated 0.1% DMSO control. Relative activation was obtained by dividing the fold activation of the tested compound at a respective concentration by the fold activation of the respective reference agonist (THRα/β: 1 μM T3, PPARα: 1 μM GW7647, PPARγ: 1 μM rosiglitazone, PPARδ: 1 μM L-165041, RORα: 10 μM SR1001, RORβ: 3 μM berberine, RORγ: 1 μM SR1001, LXRα: 1 μM T0901317, FXR: 1 μM GW4064, VDR: 1 μM calcitriol, PXR: 1 μM SR12813, CAR: 1 μM CITCO, HNF4α: 10 μM 4-hydroxy-[1,1'-biphenyl]-3-carboxylic acid^42^, RXRα: 1 μM bexarotene). Toxicity-adjusted data for resmetirom on THRα were calculated by dividing the relative activation by the relative viability obtained from the cytotoxicity assay at the corresponding concentration. Separate control experiments to exclude nonspecific reporter gene activation or VP16-mediated effects were performed following the same procedure in the absence of a nuclear receptor expression plasmid (pFR-Luc, pRL-SV40, and pECE-SV40-Gal4-VP16 only). EC_50_/IC_50_ values and the standard errors thereof were calculated with the mean fold activation values by OriginPro, Version 2025 (OriginLab Corporation, Northampton, MA, USA) fitting a dose–response curve with variable Hill slope (Levenberg–Marquardt algorithm). All assays were validated with the above-mentioned reference agonists, which yielded EC_50_/IC_50_ values in agreement with the literature.

### Cytotoxicity

HEK293T cells were seeded in DMEM high glucose, supplemented with sodium pyruvate (1 mM), penicillin (100 U/mL), streptomycin (100 μg/mL), and 10% FBS in 96‐well plates (4 × 10^4^ cells/well). After 24 h, the cells were incubated with resmetirom (final concentrations 1, 3, 10, 30, 50 and 100 μM), bexarotene (100 μM) as positive control, or 0.1% DMSO in Opti-MEM as negative control. After 24 h, the medium was aspirated, and the cells were incubated with 100 μM resazurin solution in Opti-MEM (prepared from a 0.15 mg/mL resazurin stock in Dulbecco’s phosphate-buffered saline (DPBS)) for 60 min. The metabolic activity was measured by assessing conversion of resazurin to resorufin, which was quantified by measuring absorbance at 570 nm and a reference wavelength of 600 nm on a Tecan Spark 10 M luminometer (Tecan Deutschland GmbH). Each experiment was repeated three times in duplicates.

### Target gene quantification

HepG2 cells were seeded in 6-well plates (1 × 10^6^ per well). After 24 h, medium was changed to Opti-MEM supplemented with 1% charcoal-stripped FBS, penicillin (100 U/mL), streptomycin (100 μg/mL), and L-glutamine (2 mM). After an additional 24 h, cells were incubated with the test compounds dissolved in the same medium with 0.1% DMSO or medium with 0.1% DMSO alone as untreated control for 24 h, harvested, washed with cold phosphate-buffered saline (PBS), and then directly used for the next step. Total RNA was extracted from HepG2 cells using the total RNA Mini Kit (Omega Bio-Tek, Inc.) and the same amount of RNA per sample was then reverse transcribed into cDNA with the High-Capacity cDNA Reverse Transcription Kit (Thermo Fisher Scientific, Inc.). Target gene expression was then studied by quantitative real-time PCR (qRT-PCR) analysis with a qTower^3^/G (Analytik Jena GmbH & Co. KG) using PowerSYBRGreen (Life Technologies). Each sample was set up in duplicates and repeated in four independent experiments. Data were analyzed by the comparative ΔΔC_t_ method with glyceraldehyde 3-phosphate dehydrogenase (GAPDH) as reference gene. The following primers were used (human genes): hGAPDH fwd: 5′-ATA TGA TTC CAC CCA TGG CA-3′, rev: 5′-GAT GAT GAC CCT TTT GGC TC-3′; hCYP8B1 fwd: 5′-CTG GAG ACC AAG CAG TCC TTT G-3′, rev: 5′-GAT ACT CCT GCC CAC TGG ACA T-3′; hFBP fwd: 5′-AGC CTT CTG AGA AGG ATG CTC-3′, rev: 5′-GTC CAG CAT GAA GCA GTT GAC-3′; hG6PC1 fwd: 5′-GGG AAA GAT AAA GCC GAC CTA C-3′, rev: 5′-CAG CAA GGT AGA TTC GTG ACA G-3′; hPCK fwd: 5′-CCA CAG CGG CTG CAG AAC AT-3′, rev: 5′-GAA GGG CCG CAT GGC AA-3′; UGT1A1 fwd: 5′-CCT TGC CTC AGA ATT CCT TC-3′, rev: 5′-ATT GAT CCC AAA GAG AAA ACC AC-3′.

### Lipogenesis assay

HepG2 cells were seeded in collagen G-coated 96-well plates (1.0 × 10^4^ per well) and incubated for 24 h. Then, the cells were incubated with the test compounds dissolved in culture medium with 0.1% DMSO or medium with 0.1% DMSO alone as negative control for 72 h. The cells were washed with PBS (2 × 50 μL), fixed with 10% formalin solution (50 μL) for 15 min, and washed again with H_2_O (2 × 50 μL) followed by 60% *i-*PrOH (50 μL). Neutral lipid droplets were stained with 0.3% Oil Red O solution (in 60% *i-*PrOH, 50 μL) for 10 min. After washing the cells quickly with 60% *i-*PrOH (50 μL) and PBS (2 × 50 μL), they were visualized under a BZ-X810 microscope (KEYENCE, Frankfurt, Germany) using a 40 × objective. The images were analyzed using ImageJ software (v1.54) according to a literature method^45^.

### Statistical power analysis

To determine the minimum sample size for in vitro experiments, statistical power analyses were conducted using OriginPro, Version 2025b (OriginLab Corporation, Northampton, MA, USA) and the parameters from Table 1.

**Table 1:** Statistical Power Analysis of the used assay systems.

Parameters/ Assay	Reporter gene (inactive NRs)	Reporter gene (constitutive NRs)	qPCR (upregulation)	qPCR (downregulation)	Lipogenesis
Test	two-sample t-test	two-sample t-test	two-sample t-test	two-sample t-test	two-sample t-test
Sides	one-sided	two-sided	two-sided	two-sided	one-sided
Expected mean (reference)	0.05	1	100	100	1
Expected mean (sample)	0.3	0.5	200	50	4
Standard deviation	0.03	0.05	20	20	0.5
Rejection level	0.05	0.05	0.05	0.05	0.05
Desired power	0.8	0.8	0.8	0.8	0.8
*N*	2	2	3	3	2

### Molecular docking

Molecular docking was performed using AutoDock Vina^14^, as implemented in Chimera, version 1.18^46^. The Tetrac-bound structure of PPARγ (PDB ID: 6tsg^13^) was used as template and the co-crystallized ligand was removed. The protein and ligand structures were pretreated using the “Dock prep” function with default settings, including the deletion of solvent, addition of hydrogens and Gasteiger charges. The docking grid was defined as a box around the ligand with the center coordinates (− 2.37707, − 3.0411, − 21.9747) and a dimension of (19.2015, 13.7919, 19.1988). The exhaustiveness was set to 8 and the top 10 scoring binding modes were visualized in ChimeraX, version 1.9^47^. To validate the method, Tetrac was redocked five times into the crystal structure using the identical settings, yielding low root mean square deviation (RMSD) values (mean RMSD = 0.7572 ± 0.0020 Å). Resmetirom was docked five times into the crystal structure and in all cases the pose shown in Fig. 1d (RMSD = 0.0395 ± 0.0017 Å) was found within the top 5 binding modes.

## Supplementary Information

Supplementary Information.

## Data Availability

All data generated or analyzed during this study are included in this published article (and its Supplementary Information files).
