# Supplementary material for: The metabolic dysfunction-associated steatohepatitis (MASH) drug resmetirom exhibits broad nuclear receptor activity with minimal functional impact
Source: Sci Rep. 2026 Jan 29;16:4083. doi: 10.1038/s41598-026-37494-y (PMC12855932; doi:10.1038/s41598-026-37494-y)
Supplement: Supplementary file 1 — Supplementary Information. [file 41598_2026_37494_MOESM1_ESM.pdf]

Supplementary Information

The metabolic dysfunction-associated steatohepatitis (MASH) drug resmetirom exhibits broad nuclear receptor activity with minimal functional impact

Annette Kärcher[1], Laura Isigkeit[2,3], Nils Christiaan Bandomir[1], Manfred Schubert-Zsilavecz[1], Pascal Heitel[1]*

1) Goethe University Frankfurt, Institute of Pharmaceutical Chemistry, Max-von-Laue-Str. 9, D-60438 Frankfurt, Germany
2) Goethe University Frankfurt, Institute of Pharmacology & Clinical Pharmacy, Max-von-Laue-Str. 9, D-60438 Frankfurt, Germany
3) Georg-Speyer-Haus, Paul-Ehrlich-Str. 42-44, D-60596 Frankfurt, Germany

* Correspondence to heitel@pharmchem.uni-frankfurt.de

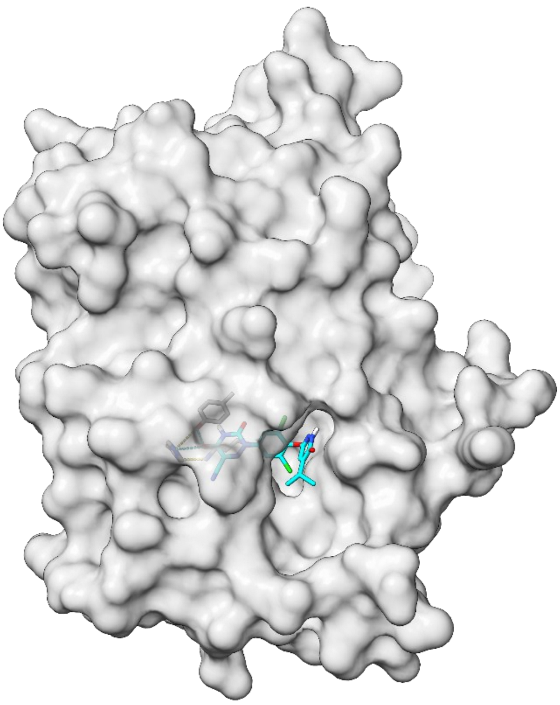

Figure S1. Molecular docking (AutodockVina) of resmetirom (cyan) into the ligand binding pocket of peroxisome proliferator-activated receptor γ (PPARγ) from PDB ID 6tsg[1]. The hydrophobic *iso*-propyl substituent is exposed to the solvent. Methodological details and a description of the binding mode can be found in the main manuscript.

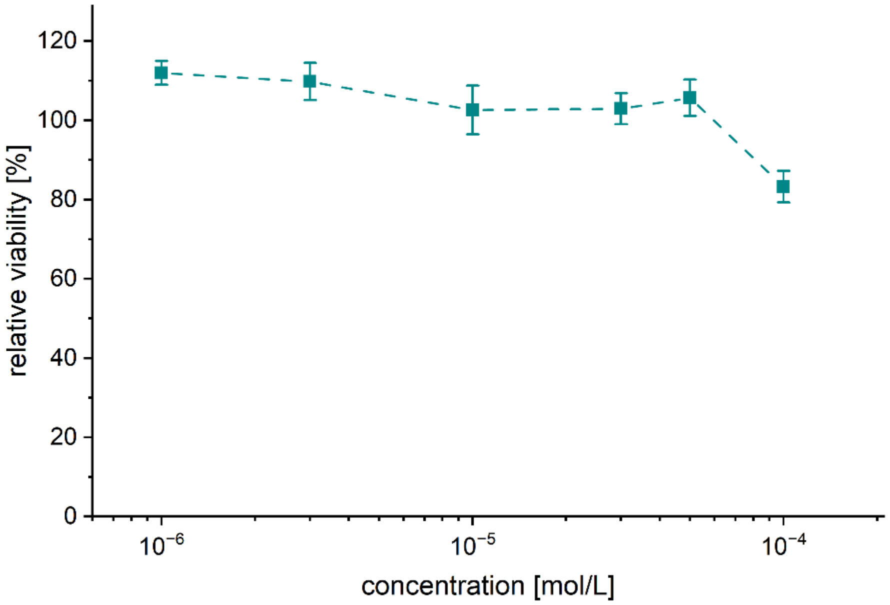

Figure S2. Cytotoxicity of resmetirom to HEK293T cells assessed by a resazurin-based metabolic activity assay. Resmetirom had no cytotoxic effect at concentrations up to 50 μM, but reduced viability at 100 μM, limiting the maximum usable resmetirom concentration for other HEK293T-based to 50 μM. Data are the mean ± SEM; $N = 3$.

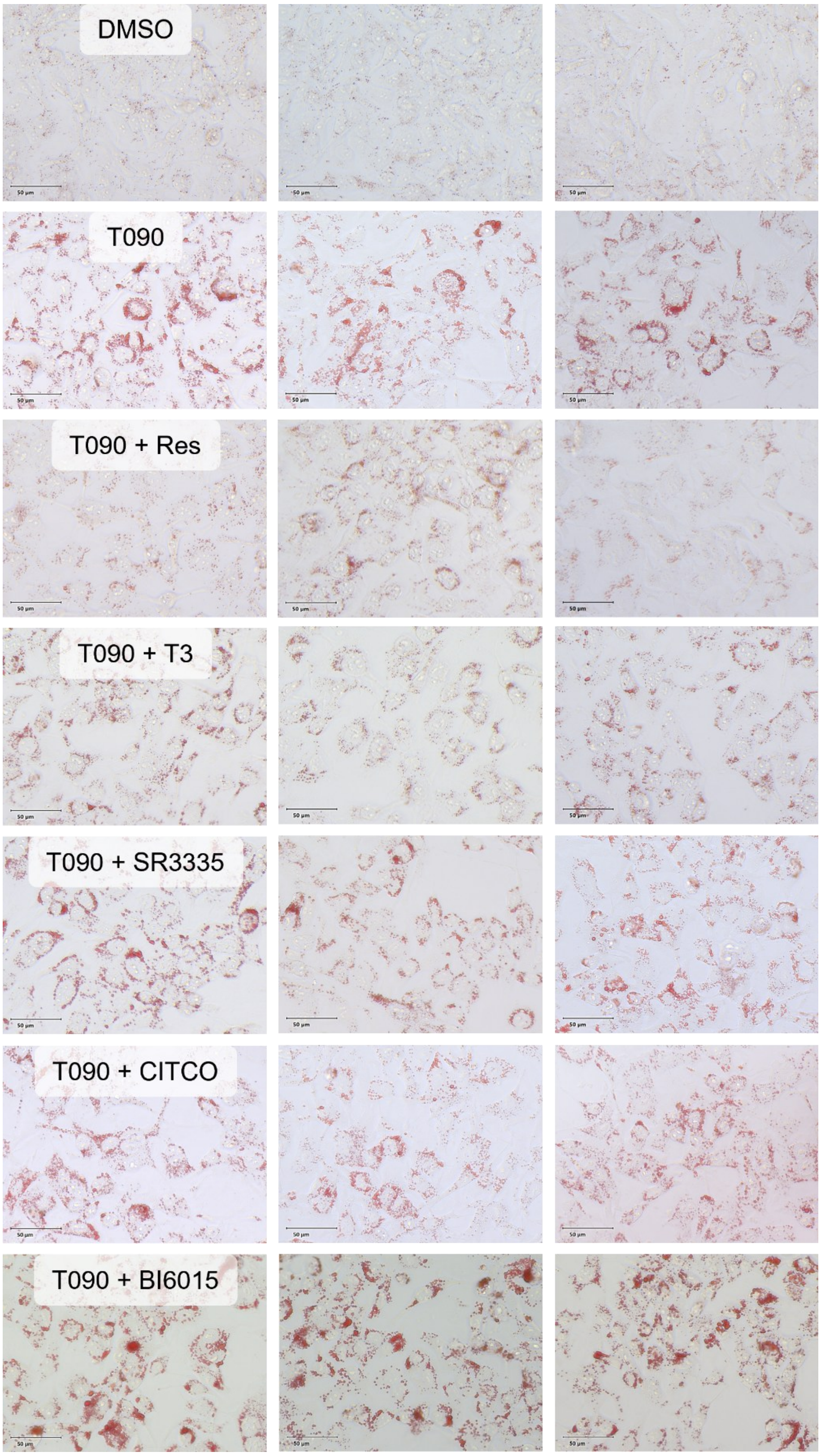

Figure S3. Full set of images from the lipid accumulation assay in HepG2 (40x magnification). After 72 h treatment with 0.1 % DMSO alone, liver X receptor agonist T0901317 (T090, 10 μM), or T090 in combination with resmetirom (Res, 30 μM), T3 (1 μM), SR3335 (10 μM), CITCO (10 μM), or BI6015 (10 μM), the cells were fixed and stained with Oil Red O. *N* = 3.

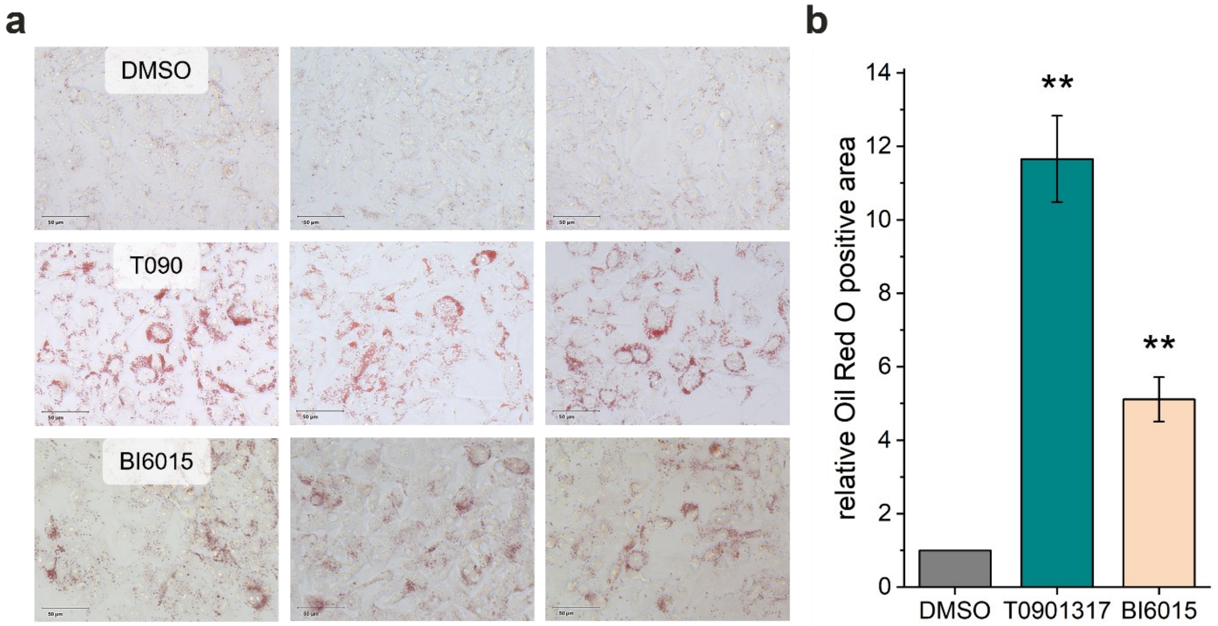

Figure S4. Lipid-droplet accumulation in HepG2 cells (Oil Red O staining) after 72 h treatment with DMSO (0.1 %) control, LXR agonist T0901317 (10 μM, T090) as positive control, or inverse HNF4α agonist BI6015 (10 μM). (a) Full set of images from three independent experiments is shown. BI6015 induced lipogenesis, but less strongly than T0901317. (b) Quantification of Oil Red O positive area relative to the 0.1 % DMSO control. Data are the mean ± SEM; $N = 3$; ** $p < 0.05$, (one-sided t-test against 0.1 % DMSO).

Supplementary Literature

1. Gellrich, L. *et al.* L-Thyroxin and the Nonclassical Thyroid Hormone TETRAC Are Potent Activators of PPARÎ. *J. Med. Chem.* **63**, 6727–6740 (2020).
